# Molecular Profiling of Atypical Tenosynovial Giant Cell Tumors Reveals Novel Non-*CSF1* Fusions

**DOI:** 10.3390/cancers12010100

**Published:** 2019-12-31

**Authors:** Theodore Vougiouklakis, Guomiao Shen, Xiaojun Feng, Syed T. Hoda, George Jour

**Affiliations:** 1 Department of Pathology, New York University Langone Health, New York, NY 10016, USA; 2 Department of Dermatology, New York University Langone Health, New York, NY 10016, USA

**Keywords:** anchored multiplex PCR, atypical tenosynovial giant cell tumor, colony-stimulating factor 1, gene fusion, RNA sequencing

## Abstract

Tenosynovial giant cell tumor (TGCT) is a benign neoplasm characterized by recurrent fusions involving the colony-stimulating factor 1 (*CSF1*) gene and translocation partners including collagen type VI alpha 3 chain (*COL6A3*) or S100 calcium-binding protein A10 (*S100A10*). Herein, we report three atypical TGCT cases with very unusual morphology comprising areas with increased cellular atypia, mitotic activity, and worrisome features that harbor unique non-*CSF1* gene fusions. Anchored multiplex PCR (AMP) for next-generation sequencing utilizing a customized panel targeting 86 cancer-related genes was performed, and it identified novel non-*CSF1*-driven gene fusions: *NIPBL-ERG*, *FN1-ROS1*, and *YAP1-MAML2*. Screening of three control TGCTs with conventional morphology found translocations involving *CSF1*, with partner genes *COL6A3*, *FN1*, and newly identified *KCNMA1*. All novel fusions were further validated by reverse transcriptase-PCR (RT-PCR) and Sanger sequencing. Late and multiple local recurrences occurred in the atypical TGCTs, while no recurrences were reported in the conventional TGCTs. Our findings reveal that atypical TGCTs harbor gene fusions not implicating *CSF1* and suggest that non-*CSF1* fusions potentially confer greater propensity to recurrences and local aggressiveness while indicating the presence of alternate pathogenic mechanisms that warrant further investigation.

## 1. Introduction

Tenosynovial giant cell tumor (TGCT), also known as giant cell tumor of tendon sheath, is a benign soft tissue lesion that is catalogued among the so-called fibrohistiocytic tumors. TGCTs are subcategorized into localized or diffuse (pigmented villonodular synovitis) variants depending on the extent of disease behavior and clinical features [1,2]. TGCTs typically affect individuals between the ages of 30 and 50 years and have an increased propensity in women [2,3]. The most common site of presentation is the fingers, and they can develop insidiously as a painless well-circumscribed growing mass. Importantly, these tumors have the proclivity to recur after excision in up to 45% of cases, thus warranting close monitoring [4]. 

A large proportion of these tumors are characterized by recurrent chromosomal translocations involving breakpoints in 1p11-13, with 2q35-37 being the most common translocation partner, while 5q22-31, 11q11-12, and 8q21-22 are less frequent [5]. Subsequent findings identified colony-stimulating factor 1 (*CSF1*) as the gene present at the 1p13 breakpoint locus and collagen type VI alpha 3 chain (*COL6A3*) gene on 2q37 as the translocation partner in a subset of these cases [6]. Interestingly, CSF1 expression was only detected in a minority of cells harboring the translocation, as demonstrated by combined fluorescent in situ hybridization (FISH) and immunohistochemistry (IHC) analysis, inferring that these are neoplastic parenchymal cells and that the translocation results in aberrant expression of CSF1 [6]. Overexpression of CSF1 in the neoplastic cell population is believed to result in abnormal recruitment of CSF1R-expressing macrophages to the tumor site [6,7]. Further evidence has demonstrated that the *CSF1* translocation is not required for CSF1 upregulation. An additional fusion partner, S100 calcium-binding protein A10 (*S100A10*), has also been identified, which replaces the 3′ untranslated region (UTR) of *CSF1* with sequences from the 3′ end of the *S100A10* gene [8]. These findings demonstrate that overexpression of CSF1 appears to play a fundamental role in the pathogenesis of TGCTs.

A minor subset of these tumors, however, exhibit unusual histologic features comprising areas with increased atypia, proliferative activity, irregular enlarged nuclei, and spindle cell morphology and are accompanied by varying degrees of fibrosis and degenerative changes. Distinction of these unconventional tumors often renders a diagnostic challenge. In the past, it has been suggested that culmination of atypical histologic features that exhibit worrisome morphology in the frank absence of sarcomatous change perhaps be referred to as “atypical TGCTs” [1]. 

In the present study, we pursued further expansion of our current understanding of atypical TGCTs at the molecular level. We identified novel non-*CSF1*-implicated gene fusions using anchored multiplex PCR (AMP) technology and subsequently validated the fusion transcripts by reverse transcriptase-PCR (RT-PCR) and Sanger sequencing. Following these findings, we further compared the transcriptome landscape of conventional TGCTs using RNA sequencing and found distinct differences among the two groups. 

## 2. Results

### 2.1. Clinical Presentation and Morphology of Atypical TGCTs

The cohort consisted of three atypical TGCTs with unconventional morphologic features comprising areas with increased cellularity, varying degrees of pleomorphism, and mitotic activity. The lesions ranged in size from 2 to 3.8 cm (mean = 3 cm) in greatest dimension and arose either on the wrists or ankle. Multiple local or late recurrences developed in patients where sufficient follow-up time was available; however, no distant metastases were identified in any patient.

**Figure 1:**
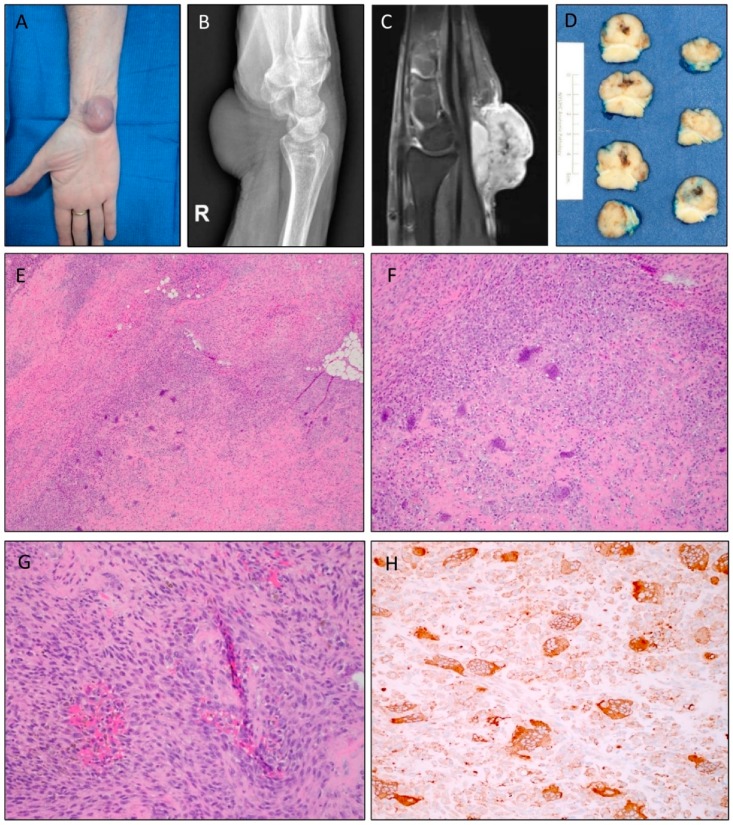
Clinical presentation, radiologic features, and histomorphology of Case 1. (**A**) A soft tissue mass was seen located on the volar aspect of the wrist. (**B**) Lateral X-ray of the right wrist revealed a nonspecific mass. (**C**) Sagittal T1 fat-suppressed postcontrast MRI showed hyperintensity of the lesion. (**D**) Gross appearance of the excised tumor demonstrated an area of central hemorrhage encased by a partial deep fibrous band. (**E**) Photomicrograph with low-power magnification showing a cellular neoplasm growing in a vague fascicular pattern with scattered osteoclast-like multinucleated giant cells and areas of hyaline matrix deposition and focal osteoid matrix formation (original magnification ×4). (**F**) Areas of hypercellularity with stromal hyalinization and osteoid deposition (original magnification ×10). (**G**) Higher-power magnification demonstrated areas of increased cellularity with spindle cells with enlarged nuclei, open chromatin, and focal enlarged nucleoli. Note the lack of necrosis (original magnification ×20). (**H**) Tumor cells displayed immunopositivity for CD68, both mononuclear and multinucleated cells.

The index case (Case 1) was from a 69-year-old female who presented with an exophytic mass located on the volar aspect of the right wrist that had increased in size and grown painful over a nine-month period (Figure 1A). A lateral X-ray of the wrist showed a large nonspecific soft tissue mass (Figure 1B), and subsequent magnetic resonance imaging (MRI) with IV contrast demonstrated a 2.7 × 2.4 × 2.2 cm diffusely enhancing mass, most consistent with an aggressive neoplasm (Figure 1C). Ultrasound-guided biopsy of the mass revealed an atypical spindle and giant cell tumor with fibrotic and collagenized areas as well as areas displaying osteoid-type early bone production. Gross examination of the marginal excision specimen revealed a well-circumscribed 3.2 × 2.7 × 1.8 cm tan-pink mass with a central hemorrhagic area partially surrounded by a deep fibrous band (Figure 1D). Microscopically, the tumor was composed of areas consisting of conventional TGCT morphology with mononuclear stromal cells, multinucleated giant cells, and histiocytes with bland cytologic features (Figure 1E). Admixed throughout the tumor were areas of increased atypia and heterogeneous growth, with varying degrees of fibrosis, hyalinization, bone formation, and degenerative changes (Figure 1F). Focal areas harboring fibrous spindle cells and prominent cellularity were also seen in addition to increased mitotic activity, but there were no atypical mitotic figures (Figure 1G). The tumor cells showed robust coarse immunopositivity for CD68 (Figure 1H) and displayed a variable Ki-67 proliferative index.

Given the unusual features and presence of the tumor at tissue edges, close clinical follow-up for recurrences was recommended. Initial workup, including a chest computed tomography (CT) scan, was negative for distant metastases. The patient had a subsequent re-excision at seven months to remove residual tumor as well as a local re-excision at 14 months to remove a recurrence. Both re-excised specimens showed similar morphologic findings, and all surgical procedures were done in the same localized area with residual or recurrent nodules occurring adjacent to the original site of tumor. The patient is alive at 24 months of follow-up since initial diagnosis, with no evidence of distant disease (negative positron emission tomography scan and chest CT scan). 

**Figure 2:**
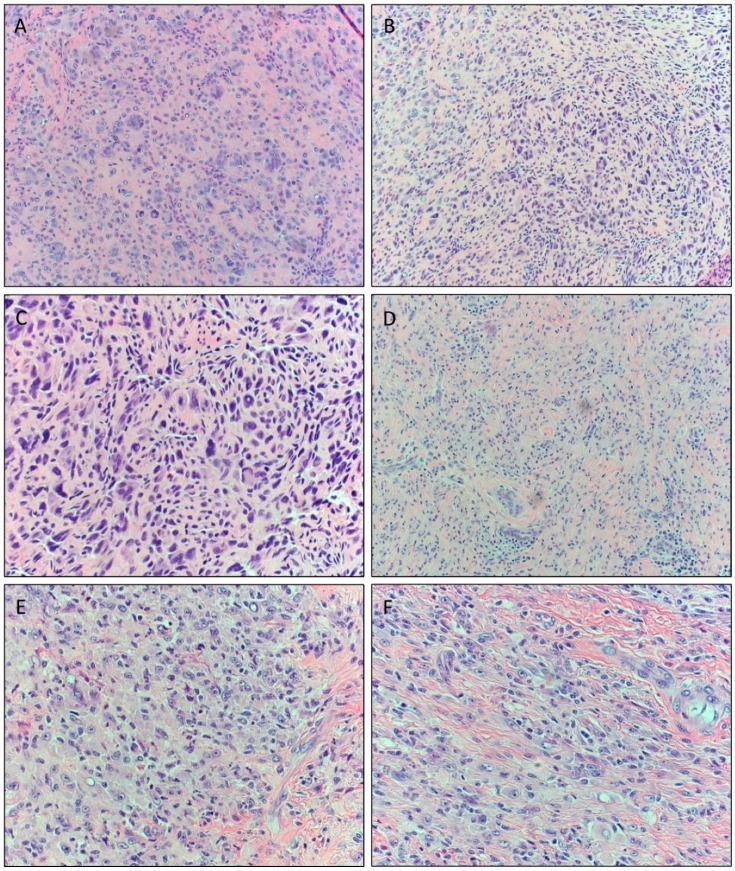
Spectrum of histomorphologic features of atypical tenosynovial giant cell tumors (TGCTs) in Case 2 (**A**–**C**) and Case 3 (**D**–**F**). (**A**) Mid-power magnification showing a cellular neoplasm with a predominance of mononucleated cells with scattered multinucleated osteoclast-like cells with intervening fibrous stroma (original magnification ×20). (**B**) Areas showing hyperchromatic pleomorphic nuclei and spindling. No necrosis was seen (original magnification ×40). (**C**) High-power magnification demonstrating multinucleated giant cells with symplastic changes in addition to increased cellular atypia in the mononuclear cell population consistent with the rendered diagnosis of atypical component within the tumor (original magnification ×40). (**D**) Photomicrograph of more benign-appearing areas composed of multinucleated giant cells and sheets of epithelioid histiocytoid cells arranged in a syncytial pattern (original magnification ×20). (**E**) Areas showing presence of larger pleomorphic nuclei with open chromatin and prominent nucleoli (original magnification ×40). (**F**) Scattered areas of cells with enlarged nuclei, spindle cells, and cells with virocyte-like nucleoli conferring worrisome morphology (original magnification ×40).

A review of archived cases of atypical TGCTs identified an additional two cases. Case 2 was from a 46-year-old female who presented with a recurrent left wrist mass that developed in the same location nearly nine years following initial excision. The tumor displayed conventional TGCT features (Figure 2A) in addition to areas of increased cellular atypia with presence of hyperchromatic nuclei (Figure 2B). Moderate morphologic atypia was present in the tumor, but some appeared to represent symplastic or degenerative atypia (Figure 2C). The Ki-67 proliferation index was <5%, suggesting a nonmalignant proliferation, and no significant mitotic activity was identified in the tumor. The recurrence was morphologically similar to the initial tumor, with a nodular configuration and foci of hyalinization, osteoid, and mature bone formation. The tumor displayed abundant giant cells and mononuclear cells, with focal areas of spindle cells.

Case 3 was from a 64-year-old female who presented with a mildly painful, slow-growing soft tissue left ankle mass. MRI revealed a partially encapsulated mixed fluid-intensity lesion overlying the distal fibula and abutting the lateral margin of the talus and calcaneus. Diffuse soft tissue edema was present, but there was no bone destruction. On excision, the mass consisted of tan-pink soft tissue that measured 3.8 × 3 × 2.5 cm. Microscopically, the lesion harbored areas with benign-appearing TGCT histology (Figure 2D); however, it additionally showed worrisome morphologic features, including enlarged nuclei with mild to moderate pleomorphism and prominent nucleoli (Figure 2E). A few areas of myxoid change, scattered spindle cells, and cells with virocyte-like nucleoli (Figure 2F) were also appreciated, but no high-grade sarcomatous component was seen in the specimen, and the mitotic activity was low. The tumor displayed immunopositivity for CD68, CD163, and a variable Ki-67 proliferation index.

### 2.2. Identification of Novel Gene Fusions in Atypical TGCTs

**Figure 3:**
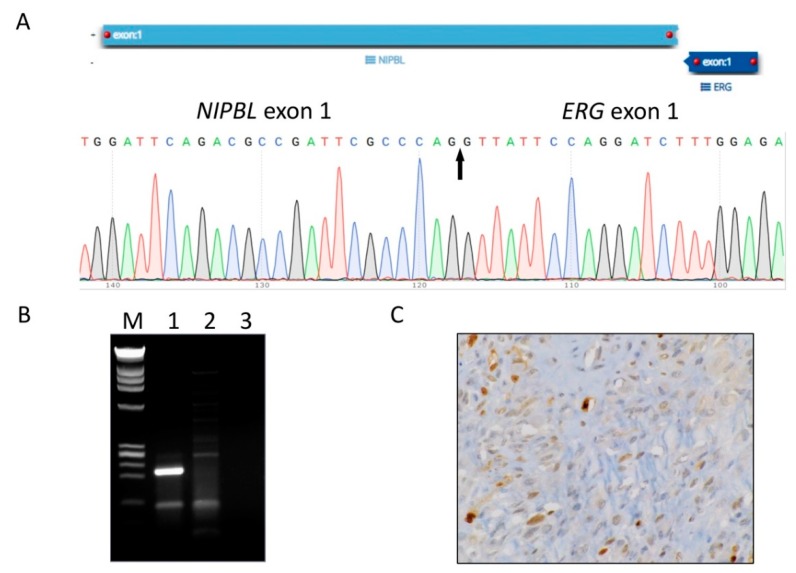

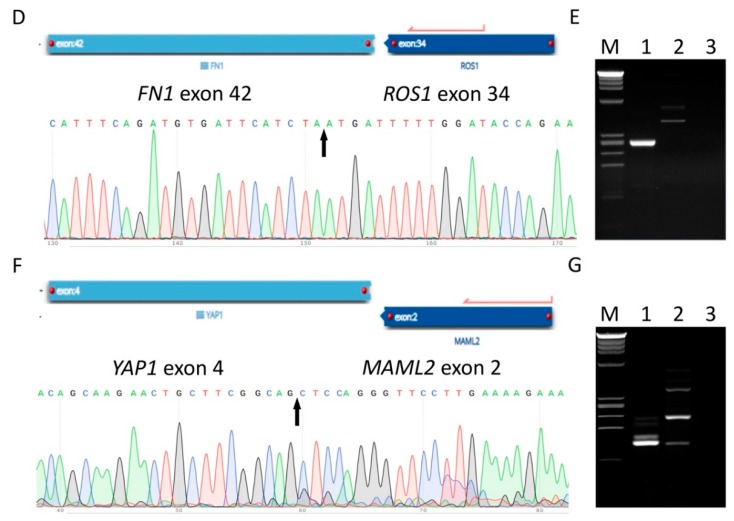
Discovery and validation of the novel gene fusions in atypical TGCTs. A partial sequence chromatogram is shown from each fusion transcript, with the arrow depicting the fusion breakpoint. Gel electrophoresis images display respective cDNA fragment amplification. A positive band can be seen in Lane 1, supporting the presence of the fusion product. M, DNA marker (Promega, Madison, WI, USA); Lane 1, patient case; Lane 2, HapMap normal RNA control; Lane 3, no template control (water control). (**A**,**B**) Identification and validation of *NIPBL-ERG* fusion transcript by anchored multiple PCR (AMP), Sanger sequencing, and RT-PCR. (**C**) ERG overexpression in the mononuclear tumor cells by immunohistochemistry (IHC). Identification and validation of *FN1-ROS1* (**D**,**E**) and *YAP1-MAML2* (**F**,**G**) fusion transcripts by AMP, Sanger sequencing, and RT-PCR.

The unconventional morphologic characteristics that were observed raised the question as to whether atypical TGCTs may potentially harbor distinct genetic aberrations. Routine FISH studies performed on the specimens were negative for traditional *CSF1* gene rearrangements. We employed in-house RNA sequencing using our customized NYU FUSIONSEQer panel, which revealed novel gene fusions not involving the *CSF1* gene. Case 1 harbored a *NIPBL* (NM_015384.4: exon: 1)/*ERG* (NM_004449.4: exon: 1) in-frame fusion in the tumor cells arising from a translocation involving loci 5p13.2 and 21q22.2, respectively. The fusion transcript was independently validated using RT-PCR, which showed a strong band in the tumor cDNA sample but not in the normal control, and by Sanger sequencing (Figure 3A,B). In addition, further IHC studies showed positive ERG expression in the mononuclear tumor cells (Figure 3C). Molecular testing on Case 2 revealed a *FN1-ROS1* gene fusion with exon 42 of *FN1* (NM_002026.2) and exon 34 of *ROS1* (NM_002944.2) (Figure 3D,E). Analogous to reported *ROS1* fusion proteins, the *ROS1* kinase domain was conserved in the case of *FN1-ROS1*. In Case 3, the fusion assay identified a *YAP1-MAML2* fusion involving exon 4 of *YAP1* and exon 2 of *MAML2* (Figure 3F,G), similar to those reported in poromas [9]. All fusion transcripts were validated by RT-PCR as well as Sanger sequencing. These findings demonstrate that atypical TGCTs harbor non-*CSF1* gene fusions.

### 2.3. Detection of New CSF1 Fusion Partners in Conventional TGCTs

**Figure 4:**
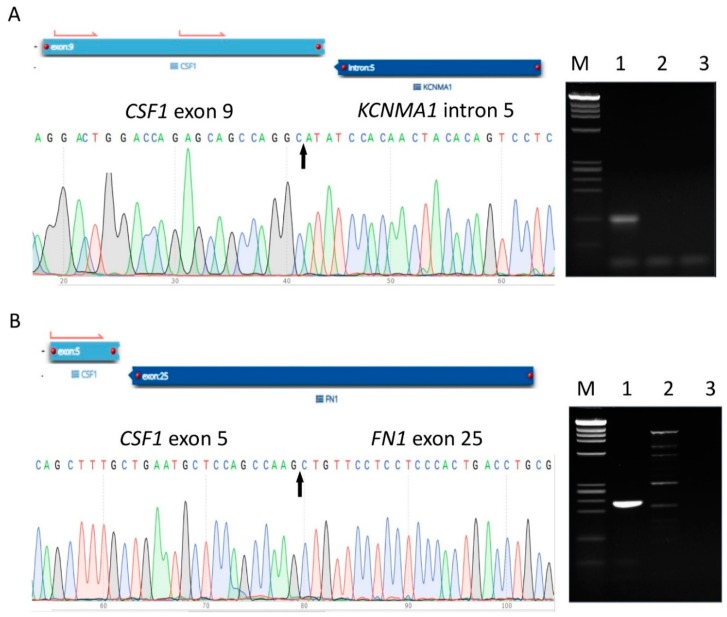
Detection and confirmation of *CSF1* partner genes identified in conventional TGCTs. A partial sequence chromatogram is shown from each fusion transcript, with the arrow depicting the fusion breakpoint. Gel electrophoresis images display respective cDNA fragment amplification. A positive band can be seen in Lane 1, supporting the presence of the fusion product. M, DNA marker (Promega, Madison, WI, USA); Lane 1, patient case; Lane 2, HapMap normal RNA control; Lane 3, no template control (water control). Identification and validation of *CSF1-KCNMA1* (**A**) and *CSF1-FN1* (**B**) fusion transcripts by AMP, Sanger sequencing, and RT-PCR.

We screened three additional TGCTs with typical morphology as a control group to compare potential differences between conventional and atypical TGCTs. All three conventional TGCTs were confirmed to have translocations involving *CSF1*, including the known *CSF1-COL6A3* and the recently reported *CSF1-FN1* [10] in addition to a new fusion partner gene *KCNMA1* (Figure 4A,B). Cases 4 and 6 showed the same exact breakpoint in exon 5 chr1:110464616, while the breakpoint was identified in exon 9 of *CSF1* in Case 5. Both of these breakpoints have been previously identified in *CSF1* fusions with partners such as *COL6A3.* The mean tumor size of the conventional TGCTs was 2.2 cm, and anatomic sites of presentation included the arm in Case 4 and the knee in Cases 5 and 6 (Table 1). None of the conventional TGCTs in our cohort showed evidence of recurrence (mean follow-up = 14.7 months). The cumulative clinical information and fusions that were identified are collectively summarized in Table 1. 

## 3. Discussion

In this study, we sought to elucidate the transcriptional signatures of atypical TGCTs and describe their morphologic and clinical correlates. Instigated by an index case, we identified novel gene fusions harbored in atypical TGCTs not involving *CSF1*. Late and multiple recurrences developed in atypical TGCT cases with sufficient follow-up in our cohort, suggesting greater propensity for local recurrence, with possible alternative mechanisms of pathogenesis. Despite the recurring nature of these tumors, unequivocal morphologic characteristics of malignancy, such as necrosis, nuclear pleomorphism, high mitotic activity, or sarcomatous change, were not present. 

To date, approximately 50 malignant TGCTs have been reported in the present literature [11]. Currently, there is no consensus pertaining to what morphologic features constitute a malignant TGCT. Criteria proposed by Enzinger and Weiss designate the malignant variant as a benign TGCT coexisting with malignant areas or a prior diagnosed benign TGCT recurring with a malignant morphology [12]. More recently, in a cohort of 10 malignant TGCTs, Al-Ibraheemi et al. demonstrated loss of CD68, CD163, and CD11c immunoreactivity in mononuclear cells with malignant morphology [11]. Furthermore, in their cohort, malignant areas displayed a mean mitotic activity of 18/10 high-power fields (HPFs). According to the WHO Classification of Tumors of Soft Tissue and Bone, malignant TGCTs in large part exhibit enlarged nuclei, ample eosinophilic cytoplasm in histiocyte-like cells, myxoid stroma, areas of necrosis, and a mitotic index of >20/10 HPFs [13]. The abovementioned accepted features of malignancy were not present in any of our cases.

The morphologic differential diagnosis of TGCT includes two important entities: giant cell tumor (GCT) of soft tissue and phosphaturic mesenchymal tumor (PMT) [14]. GCT of soft tissue is a rare mesenchymal neoplasm sharing similarities with its osseous counterpart [15]. These tumors are characterized by uniformly and evenly distributed multinucleated osteoclast-like giant cells among mononuclear cells. This finding was constantly lacking in our cohort across both conventional and atypical cases, arguing against the diagnosis of GCT of soft tissue. PMTs are rare mesenchymal neoplasms characterized by osteomalacia that is caused by a neoplasm. The hallmark of these lesions includes bland spindled cells, scattered osteoclast-like giant cells, and a basophilic matrix with flocculent calcifications [14]. None of these findings were identified in our cases. Furthermore, no patient presented with osteomalacia or systemic symptoms seen in PMTs, such as muscle pain. Our atypical cases showed significant morphologic overlapping features, which are listed in Table 1. This fact suggests that these lesions could potentially represent an unusual hitherto unrecognized entity, which merits further investigation in a larger study focusing on these particular morphologic parameters. 

ETS transcription factor ERG (*ERG*) is a protein encoding oncogene that belongs to the erythroblast transformation-specific (ETS) family of transcription factors and is frequently involved in gene fusions in prostate cancer, Ewing sarcoma, and acute myeloid leukemia [16,17,18]. Rearrangements of *ERG* and the androgen-regulated gene transmembrane serine protease 2 (*TMPRSS2*) are prevalent in a large percentage of prostate cancers, where the *TMPRSS2-ERG* fusion has been shown to correlate with prostate cancer-specific death and metastasis in men managed with expectant therapy of localized prostate cancer [19]. Several studies have linked the presence of *TMPRSS2-ERG* with dismal outlook in prostate cancer patients, suggesting that the chimeric protein serves as a poor prognostic indicator [19,20]. *ERG* rearrangements involving multiple partners have further been characterized in Ewing sarcoma. A smaller subset of these tumors lack the canonical *EWSR1-FLI1* fusion, however, have been shown to carry alternate rearrangements involving the *ERG* gene, more specifically *EWSR1-ERG* and *FUS-ERG* [21,22,23]. Aberrant expression of transcription factors as a result of these chimeric gene fusions is assumed to play crucial roles in driving neoplastic transformation. 

The breakpoints identified in our fusion are distinct from those previously described in the small round blue cell tumor/Ewing sarcoma groups [21,22,23]. A thorough literature and COSMIC database search revealed no previous reports of *NIPBL-ERG* fusions [24,25]. Furthermore, the closest reported breakpoints in *ERG* fusions to the *ERG* breakpoints identified in our case (chr21:40033588) are seen in rare variants of *TMPRSS2-ERG* (only 1.17% of the characterized and reported *ERG* fusions in prostate cancer (COSF123)). In these variants, the first observed exon in the *TMPRSS2-ERG* fusion is exon 2 of *ERG* [24,25]. Interestingly, all the described *EWSR1-ERG* and *FUS-ERG* fusions in the small round blue cell tumor/Ewing sarcoma groups involve a breakpoint situated in exons 8, 9, 10, and 11 further downstream toward the 3′ end of *ERG* [22,24,26]. Moreover, the *NIPBL-ERG* fusion in Case 1 was identified in both recurrences at 7 and 14 months, implying a common pathogenic theme for local recurrence in this specific tumor.

Cohesin loading factor (*NIPBL*) has important functions in sister chromatid cohesion by regulating the loading and unloading of cohesin onto chromatin [27]. Missense and protein-truncating mutations in *NIPBL* have been reported to cause Cornelia de Lange syndrome [28]. No *NIPBL* fusions have been reported in human cancers thus far [25]. While the biological function of the *NIPBL-ERG* fusion at present is not known, given the lack of mechanistic studies, our findings suggest that the fusion exerts its function in an analogous manner to *TMPRSS2-ERG* in prostate cancer. Although the mechanism of the *NIPBL-ERG* fusion (translocation) is different from that of *TMPRSS2-ERG* fusion (chromosomal interstitial deletion on chromosome 21), both result in overexpression of ERG under the influence of the 5′ UTR of their respective fusion partner. In this scenario, the *ERG* gene is constitutively expressed from the *NIPBL* promoter, which results in aberrant *ERG* expression. This is further corroborated by the positive immunoreactivity with ERG in Case 1. Recent preclinical studies have demonstrated that ERG inhibitory peptides (EIPs) and derived peptidomimetics bind ERG with high affinity and specificity, leading to proteolytic degradation of the ERG protein [29]. Yet, the utility of EIPs in the clinical setting remains to be investigated. 

The *FN1-ROS1* fusion identified in Case 2 is reminiscent of those encountered in *ROS1* rearranged non-small cell lung cancer (NSCLC). ROS proto-oncogene 1 (*ROS1*) is a receptor tyrosine kinase gene that is mapped to 6q22.1, and *ROS1* fusions result in protein kinase activation, which is encoded by exons 36 through 41 [30,31]. *ROS1*-rearranged tumors are found in multiple types of human cancer, including NSCLC, gastric cancer, colon cancer, and spitzoid tumors, and ubiquitous *ROS1* partner genes have been identified thus far. *ROS1* translocations are prevalent in 1–2% of NSCLCs and, more importantly, are actionable drug targets [32]. The fusion partner detected in our case, fibronectin 1 (*FN1*), encodes an extracellular matrix glycoprotein with a fibronectin type III domain. *FN1* gene fusions have been reported in PMTs, cartilaginous tumors, and more recently in calcifying aponeurotic fibromas [33,34,35]. The mechanism by which ROS1 becomes oncogenic still remains unclear to date. 

Ancillary IHC studies performed on Case 3 were positive for CD68 and CD163 and negative for EMA, Cam 5.2, AE1/AE3, ALK, Sox10, STAT6, MelanA, and p63. The tumor displayed a few areas of myxoid change and scattered cells with virocyte-like nucleoli, which raised the possibility of a myxoinflammatory fibroblastic sarcoma; however, FISH studies utilizing bacterial artificial chromosome (BAC) probes showed no additional abnormalities in *TGFBR3*, *MGEA5*, *VGLL3*, and *BRAF* genes. The *YAP1-MAML2* fusion detected in our case is seen in poromas and has been shown to augment anchorage-independent epithelial cellular growth [9].

The additional newly identified partner gene, potassium calcium-activated channel subfamily M alpha 1 (*KCNMA1*), in the conventional TGCT involved exon 9 of *CSF1* and intron 5 of *KCNMA1*. *KCNMA1* encodes calcium-activated potassium channels, and gene fusions implicating *KCNMA1* have been reported in prostate adenocarcinoma and leukemia [36]. Similar to what was reported before in *CSF1* fusions in TGCTs, the downstream amino-terminal part of *CSF1*, which interacts with its receptor CSF1R, was not encoded by the chimeric transcripts in any of the three conventional TGCTs in our cohort. This substantiates the tumorigenesis of TGCT, which depends on high level of *CSF1* expression in the neoplastic cells, which in turn will recruit the non-neoplastic CSF1R-expressing cells [8].

Besides gene fusions, recent studies have also detected the existence of additional genomic alterations involved in the pathogenesis of TGCTs. Recurrent somatic *CBL* missense mutations were observed in up to 35% of TGCTs and have been associated with increased *JAK2* expression and shorter local failure-free survival [10]. Notably, the authors emphasized the potential role of targeted therapies with JAK2 inhibitors in *CBL*-mutant cases. Loss of *CDKN2A/B* has further been reported in a case of malignant TGCT with bilateral pleural metastatic disease [37]. Given that *CDKN2A* is a crucial component of the RB1 pathway, perturbations in the tumor suppressor pathway will result with deletion of *CDKN2A* [38]. These findings, though beyond the focus of our research question, underline the molecular heterogeneity of TGCTs and provide the rationale for further exploration. 

Our study highlights novel kinase and ETS transcription factor gene driven pathways of tumorigenesis in atypical TGCTs. One caveat of our study is the limited sample size of atypical TGCTs, which is due to the rarity of its occurrence. Nonetheless, our findings suggest that atypical TGCTs may represent a distinct subtype that is different on the molecular level from conventional *CSF1*-driven TGCTs that show worrisome morphologic features and possibly greater propensity to local recurrences. 

## 4. Materials and Methods

### 4.1. Case Selection

After institutional review board (IRB) approval (s18-00637), a total of 6 cases, i.e., 3 atypical TGCTs and 3 conventional TGCTs, were selected for this study from patients diagnosed at New York University Langone Health. No patient consent was required given that there was no direct patient contact. A retrospective archival search was performed, and all available slides were reviewed by two experienced bone and soft tissue pathologists (S.T.H. and G.J.) at our institution who concurred with the diagnoses.

### 4.2. Immunohistochemistry

Immunostains were performed on a Ventana BenchMark Ultra Autostainer (Ventana Medical Systems, Tucson, AZ, USA). The following prediluted antibodies were used: anti-CD68 (KP-1) primary antibody (Ventana, Tucson, AZ, USA), and anti-ERG (EPR3864) rabbit monoclonal primary antibody (Ventana, Tucson, AZ, USA) with appropriate controls. 

### 4.3. Detection and Validation of Fusions 

FISH using specific BAC probes targeting *CSF1* were used. Subsequently, RNA was extracted and sequenced using a customized panel targeting 86 cancer-related genes (NYU FUSIONSEQer) using anchored multiplex PCR (ArcherDX^®^, Boulder, CO, USA). Briefly, RNA extraction was performed using automated Maxwell^®^ RSC RNA FFPE Kit (Promega, Madison, WI, USA) and quantified using the Qubit fluorometric assay (Thermo Fisher Scientific, Waltham, MA, USA). Library preparation was carried by two-step PCR reactions (first- and second-strand cDNA synthesis) using manufacturer-provided reagents and designed primers (ArcherDX^®^, Boulder, CO, USA). The libraries were quantified using a quantitative PCR assay (Library Quantification Kit, KAPA Biosystems, Wilmington, MA, USA) and were then pooled and sequenced via the Illumina NextSeq system (2 × 150 bp paired end sequencing). Data analysis was performed on a Health Insurance Portability and Accountability Act (HIPAA)-compliant high-performance computing system using manufacturer-provided bioinformatics pipeline. One-step RT-PCR was performed using QIAGEN OneStep RT-PCR Kit (Qiagen, Germantown, MD, USA) with primers designed based on the fusion gene: NIPBL-ERG 210F: 5′-TTATAGTCTCTCGCCACAGCG-3′ and NIPBL-ERG 210R: 5′-GTCCTTCAGTAAGCCAGCCC-3′, FN1-ROS1 278F: 5′-TGTTACCGTGGGCAACTCTG-3′ and FN1-ROS1 278R 5′-TATGCCAGACAAAGGTCAGTG-3′, YAP1-MAML2 163F: 5′-CAACTCCAACCAGCAGCAACA-3′ and YAP1-MAML2 163R: 5′-AAAGCCATTGGGTCGCTTGC-3′, CSF1-KCNMA1 120F: 5′-CTGGCCATCCTCCTGGAATG-3′ and CSF1-KCNMA1 120R: 5′-CTCTCTTTGAGGGAGGGAGGA-3′, CSF1-FN1 222F: 5′-CAGTTGCTGGAGAAGGTCAAG-3′ and CSF1-FN1 222R : 5′-AACGCACCAGGAAGTTGGTTA-3′). RT-PCR product was purified using GenElute™ PCR Clean-Up Kit (Sigma-Aldrich, St. Louis, MO, USA) and sequenced on the ABI 3500× L genetic analyzer (Applied Biosystem/ThermoFisher, Foster City, CA, USA) using the same primer sets as shown above.

## 5. Conclusions

Our data show that atypical TGCTs harbor non-*CSF1* fusions, suggesting the existence of alternate mechanisms of pathogenesis not implicating *CSF1*. This is underscored by the presence of novel fusion transcripts identified in these tumors that potentially portend increased local aggressiveness and recurrence in an otherwise benign lesion. Further investigation of our findings in a larger cohort is warranted.

## Figures and Tables

**Table 1:** Summary of clinical features and molecular findings. N/A, not applicable.

Case	Tumor Type	Fusion	Location/Laterality	Size (cm)	Sex	Age (Years)	Local Recurrence	Duration to Latest Recurrence (Months)	Follow-Up after Latest Excision/Recurrence (Months)	Atypical Features
1	Atypical TGCT	*NIPBL-ERG*	Wrist/Right	3.2	Female	69	Yes; multiple	7	9	Prominent cellularity; increased mitotic activity; spindle cells
2	Atypical TGCT	*FN1-ROS1*	Wrist/Left	2; fragmented	Female	46	Yes; multiple	104	32	Hyperchromatic nuclei; symplastic change; spindle cells
3	Atypical TGCT	*YAP1-MAML2*	Ankle/Left	3.8	Female	64	No	N/A	4	Enlarged nuclei; virocyte-like nucleoli; myxoid change; spindle cells
4	TGCT	*CSF1-COL6A3*	Arm/Left	1.5	Female	14	No	N/A	14	N/A
5	TGCT	*CSF1-KCNMA1*	Knee/Right	2.5	Male	43	No	N/A	15	N/A
6	TGCT	*CSF1-FN1*	Knee/Right	2.5	Male	29	No	N/A	15	N/A
